# Albumin - bilirubin (ALBI) versus Child-Turcotte-Pugh (CTP) in prognosis of HCC after stereotactic body radiation therapy

**DOI:** 10.1186/s13014-019-1251-y

**Published:** 2019-03-27

**Authors:** Ting-Shi Su, Hai-Ming Yang, Ying Zhou, Yong Huang, Ping Liang, Tao Cheng, Long Chen, Le-Qun Li, Shi-Xiong Liang

**Affiliations:** 1 Department of Radiation Oncology, Affiliated Tumor Hospital of Guangxi Medical University, Nanning, 530001 Guangxi Zhuang Autonomous Region China; 2 Department of Radiation Oncology, Rui Kang Hospital, Guangxi Traditional Chinese Medical University, Nanning, 530001 Guangxi Zhuang Autonomous Region China; 3 Department of Hepatobiliary Surgery, Affiliated Tumor Hospital of Guangxi Medical University, Nanning, 530001 Guangxi Zhuang Autonomous Region China

**Keywords:** Hepatocellular carcinoma, Stereotactic body radiation therapy, Child-Turcotte-Pugh, Albumin–bilirubin, Prognosis

## Abstract

**Background:**

Child-Turcotte-Pugh (CTP) score extensively used to assess hepatic function, predicting postoperative outcome of hepatocellular carcinoma (HCC) patients. Lately, the albumin–bilirubin (ALBI) grade has been identified to be a predictor of overall survival of HCC patients. In this investigation, we compared the pre-SBRT ALBI and CTP scores with the prognosis of patients with HCC.

**Methods:**

This cohort study included 594 HCC patients who treated with SBRT. Overall survival (OS) rates were measured from treatment date to death date or last follow-up. We compared ALBI score with the CTP score in predicting long-term survival.

**Results:**

The average follow-up time was 21 months (1 to 82 months). The CTP and ALBI ratings have discriminatory for long-term survival across the groups. CTP class was significantly related to OS, with a median OS of 29.9 months in CTP-A, 11.5 in CTP-B (*P* < 0.0001). ALBI grade is also significantly related to OS, with a median OS of 53.0 months in ALBI-1, 19.5 months in ALBI-2, and 6.5 months in ALBI-3(*P* < 0.0001). Within CTP-A class, CTP score-A5/A6 and ALBI grade has a similar predictive power (all *P* < 0.001). However, both CTP score and ALBI grade have no predictive power in CTP ≥ B7 class (all *P*>0.05).

**Conclusions:**

To assess liver dysfunction in HCC patients before SBRT, traditional CTP classification is a necessary but imperfect tool for assessing HCC liver injury. The ALBI score is a more objective, discriminatory and evidence-based approach in CTP-A groups, and need to be validated in CTP ≥ B7 class.

## Introduction

Hepatic function / dysfunction is closely related to the progression of hepatocellular carcinoma (HCC) and are a key determinant. To assess the severity of liver function/dysfunction, the Child-Pugh (CTP) classification has been widely used. Many HCC staging systems, such as Barcelona Clinic Liver Cancer (BCLC) staging system [1], use CTP classification as an indicator for hepatic disease severity. However, the CTP classification not only includes objective biosynthetic parameters such as circulating albumin, bilirubin and coagulation characteristics, but also more subjective parameters such as the presence and severity of ascites and encephalopathy. The clinical evaluation of ascites and encephalopathy may be affected by the lack of reproducibility among clinicians. To overcome these limitations, albumin-bilirubin (ALBI) grade is an objective method of assessing hepatic dysfunction based on albumin and bilirubin levels and has been proposed as an alternative to the CTP rating [2]. More and more studies have validated the predictive accuracy of ALBI classification for multi-region cohorts of different stage HCC and for patients undergoing different treatment, such as liver resection, radiofrequency ablation, transarterial chemoembolization and Sorafenib [3–11].

With the progress of radiation technology, stereotactic body radiation therapy (SBRT), recommended as category 2B for HCC in the version of the National Comprehensive Cancer (NCCN), has become an alternative treatment of HCC in clinical practice worldwide [12]. In this study, we predicted the long-term survival of 594 patients of HCC treated with SBRT by externally validating and comparing the ability of ALBI and CTP grading.

## Materials and methods

### Patients

From January 1, 2011 to December 31, 2016, 657 primary liver cancer patients were treated with SBRT. Before any form of definitive treatment, we comprehensively recorded baseline demographics, tumor burden, serum biochemistry, and severity of liver disease. Twenty-nine cases of intrahepatic cholangiocarcinoma, 15 cases for which complete data were lacking, and 19 cases that were lost to follow-up were excluded. All of 594 patients were retrospectively enrolled in this study. Survival of the enrolled patients was re-evaluated 1 month after SBRT and subsequently at every 3 or 6 months. Contrast-enhanced CT and/or MRI was compared at each follow-up. In addition, serum albumin, bilirubin, prothrombin time, alkaline phosphatase, hepatic AST and ALT, and alpha-fetoprotein level have been routinely examined in clinic.

### SBRT

Three or 4 gold markers of 0.8 mm in diameter were inserted into tumor tissue or the surrounding area of a tumor. Gross tumor volume (GTV) was delineated with visible lesion based on CT and/or MRI were performed 1 week later. GTV was established by 0–5 mm to cover the planning target volume (PTV). SBRT were using with CyberKnife® (Accuray Inc., Sunnyvale, CA, USA) for a few continuous days.

According to the volume of liver and various dose-volume constraints for the organs at risk, different radiation doses and fractions were recommended [13–16]. For the liver, VS15 was > 700 mL and/or V15 was < 1/3 absolute normal liver volume. SBRT doses were further optimized according to CTP score and V15 and VS10 (Table 1) [16]. For GTVs closed central porta of liver or gastrointestinal tract, more than 4 fractions were recommended. For the kidneys, the V15 was < 1/3 total volume. Similarly, for the duodenum, stomach, and small bowel, the maximum doses to 1 mL (D1 mL) were < 15Gy in 1–2 fractions or < 25 Gy in more than 3 fractions. For the spinal cord, the D1 mL was < 10 Gy in 1–2 fractions or < 15 Gy in more than 3 fractions.

**Table 1:** Recommendations for 3–5 fractions SBRT treatment

Dosimetric constraints for liver	Radiation dose for GTV
V_15_ < 21.5%, VS_10_ ≥ 621.8mL	SART: BED_10_ ≥ 100Gy
V_15_ < 33.1%, VS_10_ ≥ 416.2–621.8 mL	SBRT: EQD_2_ ≥ 74Gy
Without above conditions or CTP ≥ B7 class	SCRT: EQD_2_ < 74Gy

### Score point values calculation

CTP scored based on total bilirubin, albumin, and prothrombin time, and clinical findings of encephalopathy and ascites, and it was graded as: 5–6 points for CTP-A; 7–9 points for CTP-B; and 10–15 points for CTP-C [12]. The ALBI score was determined by the following formula: (log10 bilirubin × 0.66) + (albumin × − 0.085). The ALBI score was graded as: score ≤ − 2.60 as ALBI-1; − 2.59 to − 1.39 as ALBI-2; and score > − 1.39 as ALBI- 3 [2].

### Statistical analysis

All statistical analyses were undertaken with SPSS® version 23.0 (SPSS, Inc., Chicago, IL, USA) and Stata 15.0 (IBM, New York, NY, USA). Overall survival (OS) was defined from the date of SBRT until the date of death or last follow-up. A Kaplan–Meier curve with log-rank test was used to estimate the OS rates for different groups and Gehan-Breslow-Wilcoxon test was applied for the comparison of survival curves. Statistical significance was identified as *p* value < 0.05.

## Results

### Baseline characteristics

Demographic and clinical features of the HCC patients were summarized in Table 2. This study included 511 men and 83 women. There were 479 (80.6%) patients of CTP-A, 108 (18.2%) of CTP-B and 7(1.2%) of CTP-C. CTP-A patients included 219 of ALBI-1 and 262 of ALBI-2 patients. There were 2 of ALBL-1, 84 of ALBI-2 and 22 of ALBI-3 patients in the CTP-B group. CTP-C consisted of 1 of ALBI-2 and 6 of ALBI-3.

**Table 2:** Patient Characteristics and Correspondences between ALBI grades and CTP scores

Factor	Level	ALBI = 1	ALBI = 2	ALBI = 3	*P*-value	Test
N		219	347	28		
Gender	Male	188 (85.8%)	298 (85.9%)	25 (89.3%)	0.88	Pearson’s chi-squared
Age, mean (SD)		53.1918 (13.0342)	54.9885 (12.4969)	53.8571 (10.9636)	0.25	ANOVA
Prior-treatment	Yes	111 (50.7%)	183 (52.7%)	12 (42.9%)	0.57	Pearson’s chi-squared
Hepatitis B virus surface antigen	Positive	151 (68.9%)	248 (71.5%)	19 (67.9%)	0.97	Pearson’s chi-squared
	Negative	37 (16.9%)	53 (15.3%)	5 (17.9%)		
	Unknow	31 (14.2%)	46 (13.3%)	4 (14.3%)		
Hepatitis C virus status	Positive	2 (0.9%)	4 (1.1%)	0 (0.0%)	–	
PT, mean (SD)		12.9216 (1.08293)	13.8273 (1.52867)	16.3407 (2.93353)	< 0.001	ANOVA
Total bilirubin, mean (SD)		12.5096 (5.46543)	19.0009 (14.5374)	91.4571 (150.022)	< 0.001	ANOVA
Albumin, mean (SD)		42.1589 (3.23209)	34.6175 (3.30602)	26.2357 (2.968)	< 0.001	ANOVA
Direct bilirubin, mean (SD)		5.83333 (13.6228)	9.76974 (10.4189)	56.6179 (90.1434)	< 0.001	ANOVA
AST, mean (SD)		36.7945 (32.4975)	50.2781 (38.2566)	79.3929 (59.5373)	< 0.001	ANOVA
ALT, mean (SD)		33.5342 (26.089)	46.7867 (52.4872)	52.5 (40.2386)	0.001	ANOVA
ALBI score, mean (SD)		−2.88589 (.25688)	−2.15091 (.306301)	−1.11186 (.279759)	< 0.001	ANOVA
CTP score	5	206 (94.1%)	123 (35.4%)	0 (0.0%)	< 0.001	Pearson’s chi-squared
	6	11 (5.0%)	139 (40.1%)	0 (0.0%)		
	7	2 (0.9%)	59 (17.0%)	7 (25.0%)		
	8	0 (0.0%)	20 (5.8%)	7 (25.0%)		
	9	0 (0.0%)	5 (1.4%)	8 (28.6%)		
	10	0 (0.0%)	1 (0.3%)	3 (10.7%)		
	11	0 (0.0%)	0 (0.0%)	2 (7.1%)		
	13	0 (0.0%)	0 (0.0%)	1 (3.6%)		
BCLC stage	1	108 (49.5%)	121 (34.9%)	6 (21.4%)	< 0.001	Pearson’s chi-squared
	2	41 (18.8%)	68 (19.6%)	4 (14.3%)		
	3	69 (31.7%)	157 (45.2%)	11 (39.3%)		
	4	0 (0.0%)	1 (0.3%)	7 (25.0%)		
Tumor size, mean (SD)		5.72055 (6.40287)	6.85101 (4.83619)	6.68929 (4.26383)	0.054	ANOVA
Hemoglobin, mean (SD)		134.988 (17.6542)	119.383 (19.2401)	101.955 (22.4)	< 0.001	ANOVA
Platelet, mean (SD)		175.485 (71.4823)	172.494 (96.402)	131.5 (117.778)	0.092	ANOVA
Red blood cell, mean (SD)		4.64133 (.662971)	4.15443 (.725179)	3.47409 (.717799)	< 0.001	ANOVA
White blood cell, mean (SD)		6.19509 (2.65843)	6.41506 (3.22305)	5.82591 (2.94477)	0.57	ANOVA
AFP status (ng/mL)	Unknow	16 (7.3%)	12 (3.5%)	0 (0.0%)	0.049	Pearson’s chi-squared
	< 8	64 (29.2%)	83 (23.9%)	5 (17.9%)		
	8–200	59 (26.9%)	102 (29.4%)	13 (46.4%)		
	> 200	80 (36.5%)	150 (43.2%)	10 (35.7%)		
Total dose (Gy), median (IQR)		43 (42, 45)	42 (40, 45)	42 (39, 45)	0.13	Kruskal-Wallis
Fractions	1	3 (1.4%)	3 (0.9%)	0 (0.0%)	0.69	Pearson’s chi-squared
	2	3 (1.4%)	3 (0.9%)	1 (3.6%)		
	3	136 (62.1%)	188 (54.2%)	16 (57.1%)		
	4	57 (26.0%)	114 (32.9%)	9 (32.1%)		
	5	19 (8.7%)	35 (10.1%)	2 (7.1%)		
	6	1 (0.5%)	4 (1.2%)	0 (0.0%)		
BED10, median (IQR)		100.8 (89.7, 112.5)	94.0062 (86.1, 100.8)	94.0125 (87.9, 100.8)	< 0.001	Kruskal-Wallis
EQD2, median (IQR)		84 (74.75, 93.75)	78.3385 (71.75, 84)	78.3438 (73.25, 84)	< 0.001	Kruskal-Wallis

*Abbreviations*: *AFP* alpha fetoprotein, *ALBI* Albumin–Bilirubin, *AST* aspartate aminotransferase, *ALT* alanine aminotransferase, *BCLC* Barcelona Clinic Liver Cancer, *CTP* Child-Turcotte-Pugh, *PT* prothrombin time, *IQR* inter quartile range, *SD* standard deviation

Based on the ALBI grade, 219 (36.9%) patients were classified as of ALBI-1, 347 (58.4%) as of ALBI-2 and 28 (4.7%) as of ALBI-3. Correspondences between CTP and ALBI grades are listed in Table 1. For ALBI-1, there were 206 patients of CTP-A5, 11 of CTP-A6 and 2 of CTP-B. For ALBI-2, there were 123 patients of CTP-A5, 139 of CTP-A6, 59 of CTP-B7, 20 of CTP-B8, five of CTP-B9 and one of CTP-C10. For ALBI-3, there were 7 patients of CTP-B7, 7 of CTP-B8, 8 of CTP-B9, 3 of CTP-C10, 2 of CTP-C11 and one of CTP-C13.

### Discriminatory power of CTP or ALBI grade for long-term survival in the entire group

**Fig. 1:**
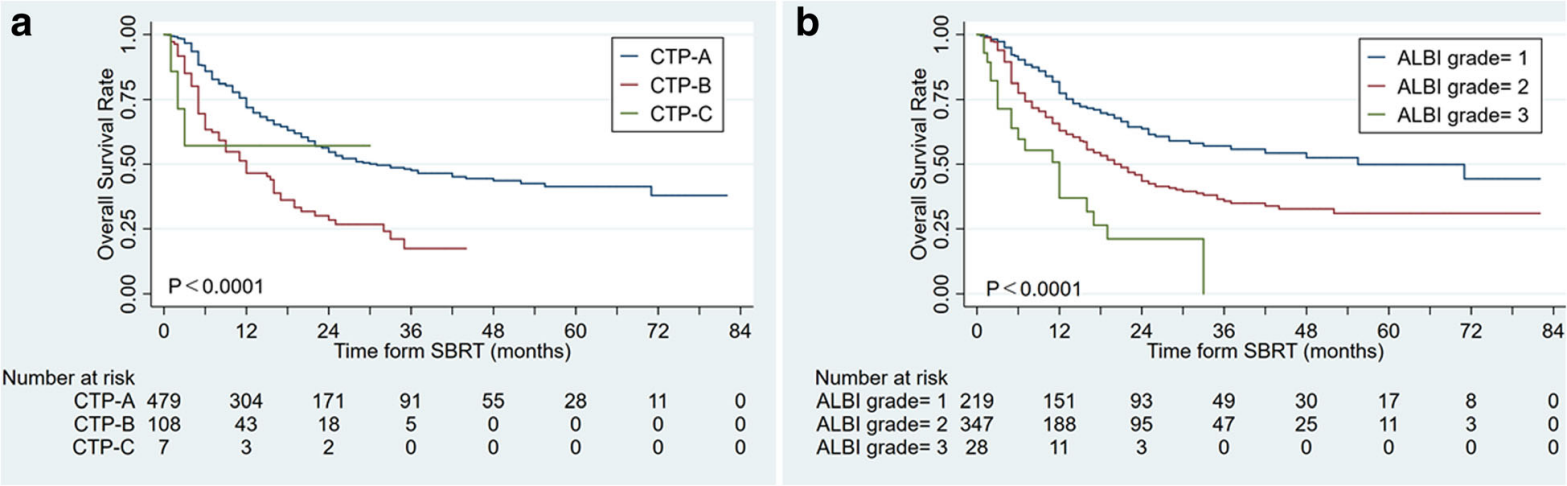
Both CTP and ALBI grade have 3 separate curves for long-term survival post-SBRT: **a** CTP classification, **b** ALBI grade

After follow up at October 18th, 299 patients had died. The follow-up time was 1 to 82 months (median, 21 months). Both CTP and ALBI grade have three separate curves for long-term survival post-SBRT. CTP class was significantly related to OS, with a median OS of 29.9 months in CTP-A, 11.5 in CTP-B. The 1-, 3-, 5- years OS was 71.9, 47.6, and 41.4% in CTP-A, respectively; the 1-, 3-years OS was 46.5 and 17.5% in CTP-B, respectively (log-rank, *P* < 0.0001, Fig. 1a). ALBI grade was also significantly related to OS, with a median OS of 53.0 months in ALBI-1, 19.5 months in ALBI-2, and 6.5 months in ALBI-3. The 1-, 3-, 5- years OS was 77.4, 57.6, and 49.9% in ALBI-1; 63.0, 35.7, and 31.0% in ALBI-2; 37.0, and 0% and 0% in ALBI-3 group, respectively (log-rank, *P* < 0.0001; Fig. 1b).

### Both CTP score with ALBI grade have the similar predictive power in CTP class a group

**Fig. 2:**
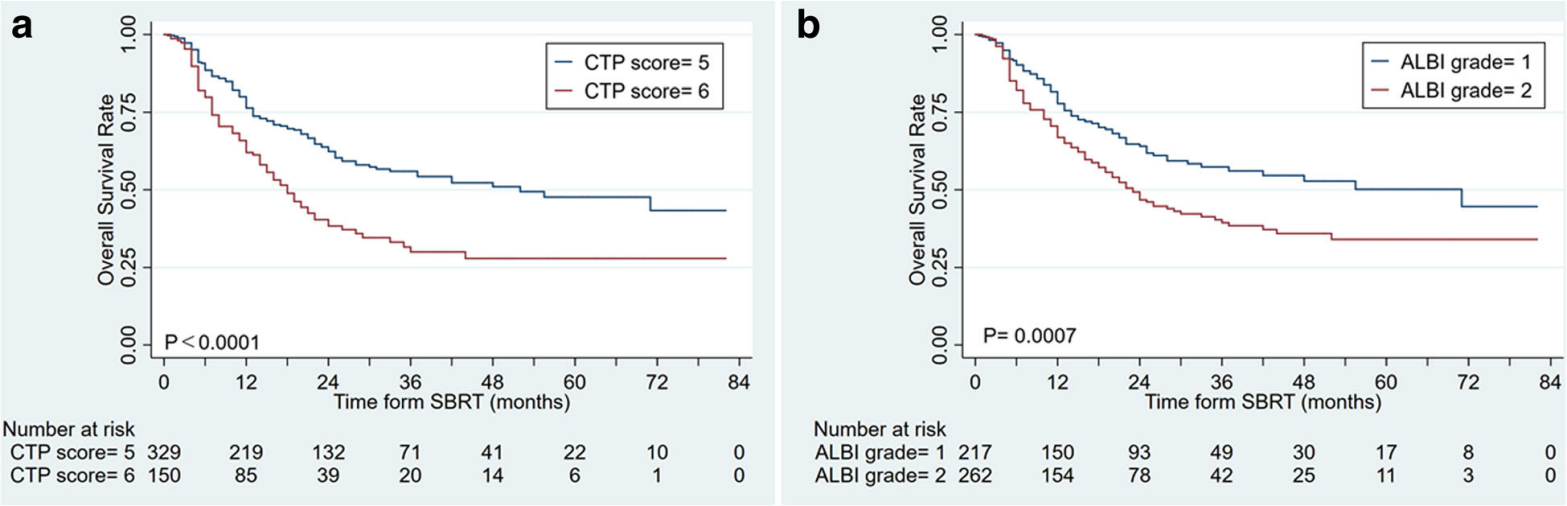
Subgroup analysis in CTP class A group: **a** CTP classification, **b** ALBI grade

For CTP-A population, A significant difference in OS was observed between CTP- A5 and A6, with a median OS of 51.0 months and 16.2 months (log-rank, *P* < 0.0001, Fig. 2a), respectively. For ALBI grade, ALBI − 1 patients had a significantly longer survival than that of ALBI − 2, with a median OS of 55.0 months and 21.9 months, respectively (log-rank, *P* = 0.0007, Fig. 2b).

### Both CTP score with ALBI grade have no predictive power in CTP ≥ B7 group

**Fig. 3:**
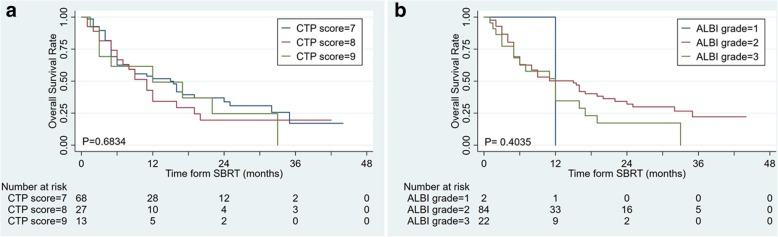
Subgroup analysis in CTP ≥ B7-B9 group: **a** CTP classification, **b** ALBI grade

For the CTP B7-B9 population, no significant change was observed in OS between in CTP scores, respectively (*P* = 0.6834, Fig. 3a). ALBI grade has no predictive power in this group, but the curves of ALBI grade tend to separate (*P* = 0.4035, Fig. 3b).

## Discussion

The key issue in predicting HCC outcomes is the liver function reserve. The traditional CTP rating system is an alternate for the severity of cirrhosis in patients with HCC. Previous studies with smaller sample tried to assess the ALBI grade for patients treated with SBRT, but lack of long-term outcome [17–19]. In this larger sample study of SBRT for HCC, we found that both CTP and ALBI grade have 3 separate curves for long-term survival post-SBRT. In CTP-A population, there was similar predictive power between CTP and ALBI scoring system. But there was no significant change between CTP-B and CTP-C groups based on CTP classification system and ALBI grade. The results in CTP ≥ B7 group need to be validated by larger sample study.

CTP classification semi-quantitative assessment included five common clinical and laboratory indicators and they are ascites, degree of hepatic encephalopathy, coagulation, serum albumin, bilirubin levels. CTP scores are commonly used in patients with cirrhosis to determine prognosis. It is subject to equal weighting of 5 parameters and any cut-off value. In addition, clinical assessment of ascites and hepatic encephalopathy may be subjective and difficult to be consistently scored by different evaluators. The ALBI score system was recently developed to assess hepatic functional reserve in HCC patients. The results of albumin and bilirubin can be readily obtained as a routine blood test, so both scores are completely objective measures of liver dysfunction. More and more studies have validated the predictive accuracy of ALBI classification for multi-region cohorts of different stage HCC and for patients undergoing different treatment, such as liver resection, radiofrequency ablation, transarterial chemoembolization and Sorafenib [3–11]. We confirmed that both CTP and ALBI systems were able to prognose HCC patients into three groups, and the application of ALBI was also verified in hepatocellular carcinoma with SBRT.

SBRT can target the tumor area more accurately. Hepatic SBRT is increasingly used in clinic. Our previous studies showed that CTP-B was significantly associated with worse OS in small (longest diameter ≤ 5 cm) HCC group [14]. We further compared long-term survival after SBRT with liver resection for small HCC with CTP- A cirrhosis, the 5-year OS was 70.0% in the SBRT group and 64.4% in the liver resection group [13]. In current study of SBRT for HCC without differentiation of tumor size and staging, the 5-year OS was 41.4% in CTP-A groups and 49.9% in ALBI-1 groups. The long-term survival data may support the clinical application of SBRT in future.

The limitations of our study are: 1. referral bias may exist in CTP ≥ B7 in this study. The number of patients with CP-B was 108 but the number of patients with CP-C was only seven. These differences among the groups was too large to achieve any statistical significance. The results need to be validated by other research groups; 2. This study is a single-center retrospective study conducted in hepatitis B endemic areas in China, whether these dosimetric data are fully applicable to patients with other risk factors for HCC is unclear.

## Conclusions

CTP assessment of HCC liver function reserve is commonly used with semi-quantitative assessment. We recommend that the ALBI grading is an alternative assessment of liver function in HCC treated with SBRT in CTP-A groups. This method is objective, discriminatory and evidence-based, with more clinical feasibility and superior prognosis, especially for patients with minimal liver dysfunction and patients receiving more active intervention. Applying these objective models to the current HCC staging system to further improve its predict ability is critical.

## Abbreviations

ALBI: Albumin–bilirubin
ALT: Alanine aminotransferase
AST: Aspartate aminotransferase
BCLC: Barcelona Clinic Liver Cancer
BED: Biologically effective dose
CT: Computed tomography
CTP: Child-Turcotte-Pugh
EQD_2_: Equivalent dose in 2 Gy fractions
GTV: Gross tumor volume
HCC: Hepatocellular carcinoma
MRI: Magnetic resonance imaging
OS: Overall survival
PTV: Planning target volume
RT: Radiation therapy
SART: Stereotactic ablative radiotherapy
SBRT: Stereotactic body radiotherapy.
SCRT: Stereotactic conservative radiotherapy
VSx: The absolute liver volume (mL) spared from x Gy
Vx: The percentage of normal liver volume receiving x Gy
